# Genome-Wide Association Study of Grain Size Traits in *Indica* Rice Multiparent Advanced Generation Intercross (MAGIC) Population

**DOI:** 10.3389/fpls.2020.00395

**Published:** 2020-04-24

**Authors:** Kimberly Ponce, Ya Zhang, Longbiao Guo, Yujia Leng, Guoyou Ye

**Affiliations:** ^1^CAAS-IRRI Joint Laboratory for Genomics-assisted Germplasm Enhancement, Agricultural Genomics Institute in Shenzhen, Chinese Academy of Agricultural Sciences, Shenzhen, China; ^2^State Key Laboratory for Rice Biology, China National Rice Research Institute, Hangzhou, China; ^3^Strategic Innovation Platform, International Rice Research Institute, Metro Manila, Philippines

**Keywords:** grain size, association mapping, MAGIC population, rice, single nucleotide polymorphism

## Abstract

Rice grain size plays a crucial role in determining grain quality and yield. In this study, two multiparent advanced generation intercross (MAGIC) populations, DC1 and BIM, were evaluated for grain size across three environments and genotyped with 55K array-based SNP detection and genotype-by-sequencing (GBS), respectively, to identify QTLs and SNPs associated with grain length, grain width, grain length–width ratio, grain thickness, and thousand grain weight. A total of 18 QTLs were identified for the five grain size-related traits and explained 6.43–63.35% of the total phenotypic variance. Twelve of these QTLs colocalized with the cloned genes, *GS3*, *GW5/qSW5*, *GW7/GL7/SLG7*, and *GW8/OsSPL16*, of which the first two genes showed the strongest effect for grain length and grain width, respectively. Four potential new genes were also identified from the QTLs, which exhibited both genetic background independency and environment stability and could be validated in future studies. Moreover, the significant SNP markers identified are valuable for direct utilization in marker-assisted breeding to improve rice grain size.

## Introduction

Grain size is one of the key agronomic traits that proceeded from unconscious selective pressure over the course of rice domestication. Improving grain size as an early adaptive response to rice cultivation is a result of unintentional selection for seeds that could survive even with deeper soil cultivation (Purugganan and Fuller, 2009). Interestingly, it was further subject to deliberate selection and breeding as it impacts rice grain quality and yield *per se* (Tan et al., 2000; Lu et al., 2013). A wide range of grain size characteristics therefore exists in modern-day rice varieties, which mainly defines consumers’ preferences and market value (Fitzgerald et al., 2009).

Rice grain size is characterized by a combination of grain length (GL), grain width (GW), and grain thickness (GT) (Tan et al., 2000; Xing et al., 2002). It is closely associated with grain weight, a major yield component along with the number of panicles per plant and the number of grains per panicle. Many QTL mapping studies using populations derived from various biparental crosses have been conducted. Over 400 QTLs for grain size have been mapped across 12 chromosomes of rice, of which 109, 95, and 107 were associated with GL, GW, and thousand grain weight (TGW), respectively (Huang et al., 2013). A few of these QTLs have been fine-mapped including *gw3.1* (Li et al., 2004), *qGL3-a* (Wan et al., 2006), *gw8.*1 (Xie et al., 2006), *gw5* (Wan et al., 2008), *GW1-1* and *GW1-2* (Yu et al., 2008), *gw9.1* (Xie et al., 2008), *GW3* and *GW6* (Guo et al., 2009), *qGL7* (Bai et al., 2010), *qGL4b* (Kato et al., 2011), *tgw11* (Oh et al., 2011), *qSS7* (Qiu et al., 2012), *qGS7* (Shao et al., 2012), *qGRL1* (Singh et al., 2012), and *GS2* (Zhang et al., 2013). Recent advances in rice functional genomics facilitated the cloning and functional characterization of several genes that either positively or negatively regulates grain size. Negative grain size regulators that were previously cloned include *GS3* (Fan et al., 2006), *GW2* (Song et al., 2007), *TGW6* (Ishimaru et al., 2013), *GW7/GL7/SLG7* (Wang et al., 2015a, c; Zhou et al., 2015), *qGL3/qTGW3* (Ying et al., 2018), and *LARGE8* (Xu et al., 2018); whereas positive grain size regulators include *GW5/qSW5* (Shomura et al., 2008; Weng et al., 2008), *GS5* (Li et al., 2011), *GW8/OsSPL16* (Wang et al., 2012b), *GS2* (Duan et al., 2015; Hu et al., 2015), *BG1* (Liu et al., 2015), *WTG1* (Huang et al., 2017; Liu et al., 2018), *GS9* (Zhao et al., 2018), and *qLGY3*/*OsLG3b* (Yu et al., 2018). It is noteworthy that these cloned genes control grain size by altering cell proliferation and/or cell expansion affecting cell numbers either in latitudinal or longitudinal directions. Furthermore, functional characterization revealed a variety of different proteins involved in a range of signal transduction pathways affecting grain size. However, only a few genes/QTLs are directly useful in breeding. As most of the genetic mapping studies conducted have relied on traditional linkage mapping using populations derived from biparental crosses, the identified QTLs are often not transferable to other genetic backgrounds since the estimated effects are limited to the two parents under study. Given the tiny fraction of the total variation present, different biparental populations yield different QTLs and with varying effects due to epistasis, pleiotropy, and QTL-by-environment interaction.

The genome-wide association study (GWAS) overcomes the limitation of biparental linkage mapping by taking advantage of many years of historical and evolutionary recombination to localize QTL in genetically diverse populations. In rice, the GWAS has been demonstrated to be a powerful complementary strategy to the biparental linkage mapping for grain size (Si et al., 2016; Duan et al., 2017; Yu et al., 2017; Ma et al., 2019). However, the GWAS using natural populations is often associated with complicated population structure and cryptic kinship, which results to spurious marker trait associations (MTAs). The use of multiparent advanced generation intercross (MAGIC) populations offers an alternative approach to the GWAS using natural populations and to linkage mapping using populations derived from biparental crosses. MAGIC has more allelic and phenotypic diversity ensuring that more QTLs segregate within the population (Cavanagh et al., 2008; Huang et al., 2015). Furthermore, it has a better control over population structure and kinship, and has been proven effective in identifying major genes via association mapping (Bandillo et al., 2013; Meng et al., 2016, 2017; Descalsota et al., 2018; Ogawa et al., 2018; Ponce et al., 2018).

In this study, association mapping was conducted in two MAGIC populations tested across three environments for 2 years to identify QTLs associated with the grain size-related traits—GL, GW, grain length–width ratio (GLWR), and TGW. The results in this study could provide valuable information to further elucidate the genetic basis of rice grain size and in marker-assisted breeding.

## Materials and Methods

### Association Mapping Panel

Two MAGIC populations developed at the International Rice Research Institute (IRRI) were used: (1) a four-parent MAGIC population, DC1, previously characterized by Meng et al. (2016), and (2) an eight-parent MAGIC population reported by Bandillo et al. (2013), which we referred to as Bandillo *indica* MAGIC (BIM) population in this study. The parents used to develop both populations were presented in Supplementary Table 1. A total of 221 and 378 lines in the DC1 and BIM populations, respectively, were evaluated for five grain size-related traits.

### Phenotyping and Phenotypic Analysis

The field trials were conducted in three testing environments, one in the Philippines at the headquarters of the IRRI, Los Baños, Laguna in 2017 and two in China at Henan in 2018 and at Hainan in 2017 and in 2018. Trials in each testing environment were laid out in an incomplete block design. Freshly harvested paddy was dried to moisture content of 12–14% and equilibrated in paper bags at room temperature for 3 months prior to measurement of grain size-related traits. GL (mm) and GW (mm) were evaluated using grain scanner (Seiko Epson, Suwa City, Japan). Grain images (0.042433 mm/pixel) were analyzed using the SmartGrain software program (Tanabata et al., 2012). GLWR was calculated as the ratio of GL and GW. GT (mm) and TGW (g) were measured according to the National Rice Grain Quality Assessment Standard of China (GB/T17891-1999). About 100 grains for each entry were evaluated for GL and GW, and a total of 6 grains were measured for GT.

The population size of DC1 and BIM varied greatly in all the three testing environments (Supplementary Table 2). Phenotypic analysis was conducted using a linear mixed model to properly handle unbalanced data. The best linear unbiased estimates (BLUEs) of each line were obtained using the PBTools (bbi.irri.org). Trait correlations were calculated and plotted using the corrplot package in R.

### SNP Genotyping

The DC1 population was sequenced with a high-density array-based SNP platform using the 55K Affymetrix Axiom Rice Genotyping Array at the CapitalBio Technology Beijing, China (Meng et al., 2017), whereas the BIM population was sequenced with a GBS approach using Illumina HiSeq at the Cornell University (Bandillo et al., 2013). A stringent filtering strategy was conducted to choose high-quality SNPs for association. All heterozygous markers were set to missing. Markers with minor allele frequency (MAF) < 0.05 were removed. Highly correlated markers (*r*^2^ > 0.95) were also excluded from the SNP data set (Supplementary Datasheet 1).

### Population Structure and Linkage Disequilibrium Analysis

Principal component analysis (PCA) was used to infer population structure. The first two principal components (PCs) were plotted using ggplot2 in R to visualize the dispersion of DC1 and BIM lines. The LD analysis was performed by pairwise comparisons in a set of filtered SNP markers (MAF < 0.05) using the LD function in TASSEL v.5.2.19 (Bradbury et al., 2007). Squared allele frequency correlations (*r*^2^) between marker pairs were used to estimate LD. The loci with significant LD were identified based on *p* < 0.0001; the rest were considered not informative. Significant intrachromosomal *r*^2^ values were plotted against physical distance, and a smoothering second degree LOESS curve (Cleveland, 1979) was fitted using the ggplot2 package in R (Wickham, 2016). The intersection of the loess curve and the critical *r*^2^ value beyond which LD was likely to be caused by genetic linkage was considered as the estimated extent of LD decay (Breseghello and Sorrells, 2006).

### Association Analysis

Association analysis was carried out using the GAPIT in R implementing a mixed linear model to account for both population structure and kinship (Yu et al., 2006). The threshold value was set at −log10(P) < 4 as shown in the Manhattan plot to identify the peak association signals. The plots were visualized using the qqman package in R (Turner, 2014). Peaks exhibiting the significance threshold level within a physical distance of ∼2.25 and 1.70 Mb for DC1 and BIM populations, respectively, were considered as a single QTL.

A promising QTL was considered when many SNPs lined up near the peak of the QTL and when it showed both genetic background independency and environment stability. Candidate genes in the promising QTLs were searched in the MSU Rice Genome Annotation database (http://rice.plantbiology.msu.edu/). Potential new genes were then selected based on the following criteria: (1) with significant MTAs accounting for over 10% of the total phenotypic variance; and (2) with significant MTAs corresponding to non-synonymous SNPs in the coding region of the genes.

## Results

### Phenotypic Variation and Trait Correlation

**FIGURE 1:**
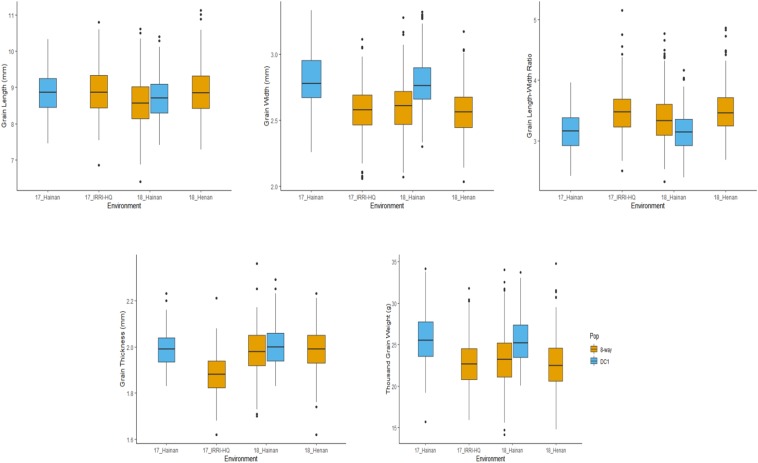
Box plot for the five grain size-related traits of MAGIC population in different environments. 17_Hainan: 2017 in Hainan. 17_IRRI-HQ: 2017 in IRRI. 18_Hainan: 2018 in Hainan. 18_Henan: 2018 in Henan.

A wide range of values for all the grain size-related traits were observed in both the DC1 and BIM populations including their founder lines across the three testing environments. Wider phenotypic variability was observed in the BIM than in the DC1 population for all the measured traits attributed to the wider phenotypic variation of the BIM founder lines (Supplementary Table 1). Most of the traits appeared to be normally distributed; however, some trait–environment combinations showed skewed distributions as evidently shown by the lopsided boxplot. Prominent data skewness was observed for GW of DC1 and BIM populations tested in Hainan 2017 and Hainan 2018, respectively (Figure 1).

**FIGURE 2:**
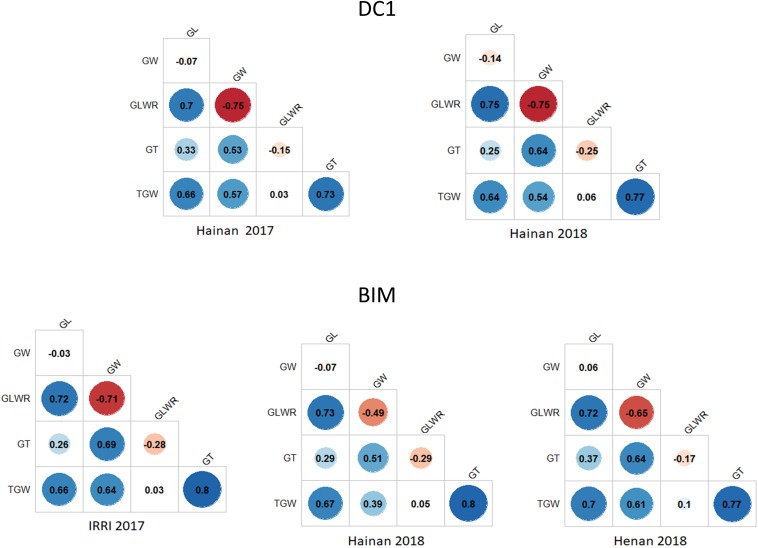
Trait correlations for the five grain size-related traits in DC1 and BIM populations tested across different environments.

The correlations of TGW with GL, GW, and GT in both populations across different environments were positive ranging from moderate (0.39) to strong (0.80). GT was negatively correlated with GLWR with correlations ranging from −0.15 for the DC1 in Hainan 2017 to −0.29 for the BIM in Hainan 2018. On the contrary, the correlations of GT with GL and GW in both populations and across different environments were positive with low to moderate values (Figure 2). The correlation between GLWR and GL was consistently high in all the trials with values ranging from 0.70 to 0.75. Negative and strong correlation (−0.75) was found between GLWR and GW in the Hainan 2017 and Hainan 2018, whereas for the BIM, it was moderate (−0.49) to strong (−0.71) in Hainan 2018 and IRRI 2017 trials, respectively.

### Population Structure and Whole Genome Pattern of LD Decay

The first two PCs, which accounted for most of the variation, were plotted to observe any subgroups in both populations under study. Results showed two subgroupings in the DC1 as previously reported by Meng et al. (2016), whereas no subgrouping was observed in the BIM population (Supplementary Figure 1).

The decay of LD along with physical distances was computed for both the DC1 and BIM populations. A critical value of the determination coefficients *r*^2^ > 0.2 was used to be the appropriate threshold for LD (Meng et al., 2016). A scatter *r*^2^ against physical distance showed a clean pattern of LD decay in the DC1 and BIM populations. The decline of LD to 50% of its initial value was at 2.25 Mb for the DC1 (Meng et al., 2016) and at 1.70 Mb for the BIM population (Supplementary Figure 2)

### QTLs Identified by Association Analysis

**FIGURE 3:**
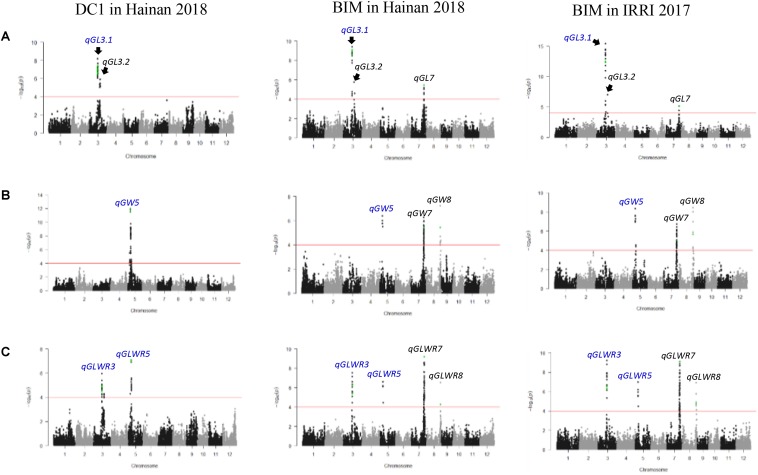
Genome-wide Manhattan plots for **(A)** grain length, **(B)** grain width, and **(C)** grain length–width ratio in DC1 and BIM measured in two testing environments. QTLs in blue color exhibited genetic background independency and environment stability. Dots in green color are SNPs corresponding to previously cloned genes (*GS3*, *GW5/qSW5, GL7*/*GW7*/*SLG7*, and *GW8/OsSPL16* on chromosomes 3, 5, 7, and 8, respectively).

A total of 329 and 334 significant MTAs were identified for the DC1 population in Hainan 2017 and Hainan 2018, respectively. For the BIM population, a total of 480, 413, and 254 significant MTAs were identified in IRRI 2017, Hainan 2017, and Henan 2018, respectively (Supplementary Table 3). These significant MTAs were delineated into a total of 18 QTLs for the five measured traits (Table 1). The genome-wide Manhattan plots for grain size-related traits with QTLs that showed genetic background independency and environment stability were presented in Figure 3. Manhattan plots for all the trait–environment combinations were presented in Supplementary Figure 3.

**TABLE 1:** QTLs identified from DC1 and BIM populations for the five grain size-related traits.

QTL	Panel	Env^a^	Year	Chr^b^	Peak SNP	Pos^c^	Alleles^d^	*p*-value	%PVE^e^	Effect^f^	Known gene
*qGL3.1*	DC1	Hainan	2017	3	AX-154083640	16806335	A/C	1.93E-09	54.73	0.3293	*GS3*
	DC1	Hainan	2018	3	AX-154083640	16806335	A/C	6.58E-09	55.28	0.0462	*GS3*
	BIM	IRRI	2017	3	rs3_16842769	16842769	C/T	3.80E-16	35.07	0.3425	*GS3*
	BIM	Hainan	2018	3	rs3_16842769	16842769	C/T	4.04E-10	21.60	0.0127	*GS3*
	BIM	Henan	2018	3	rs3_16729992	16729992	G/C	5.37E-10	21.56	0.2809	*GS3*
*qGL3.2*	DC1	Hainan	2017	3	AX-165099081	21547035	C/T	8.71E-06	54.73	0.2209	–
	DC1	Hainan	2018	3	AX-115865890	21602538	C/T	1.19E-06	55.28	0.0330	–
	BIM	IRRI	2017	3	rs3_20691702	20691702	G/T	1.01E-07	35.07	−0.1906	–
	BIM	Hainan	2018	3	rs3_20691702	20691702	G/T	1.73E-06	21.60	−0.0154	–
	BIM	Henan	2018	3	rs3_20691702	20691702	G/T	1.66E-06	17.74	−0.2016	–
*qGL7*	BIM	IRRI	2017	7	rs7_24645555	24645555	A/G	6.82E-06	35.07	0.2911	*GL7*/*GW7*/*SLG7*
	BIM	Hainan	2018	7	rs7_24669663	24669663	C/G	6.69E-06	21.60	−0.0117	*GL7*/*GW7*/*SLG7*
*qGW5*	DC1	Hainan	2017	5	AX-165092179	5364311	G/T	3.08E-15	59.78	−0.1506	*GW5/qSW5*
	DC1	Hainan	2018	5	AX-165092179	5364311	G/T	1.03E-12	24.88	−0.0021	*GW5/qSW5*
	BIM	IRRI	2017	5	rs5_4753775	4753775	A/G	4.54E-09	22.78	0.1164	–
	BIM	Hainan	2018	5	rs5_4882721	4882721	A/T	4.08E-07	17.96	−0.0231	–
	BIM	Henan	2018	5	rs5_4685451	4685451	C/G	1.37E-05	10.42	−0.0983	–
*qGW7*	BIM	IRRI	2017	7	rs7_24380529	24380529	A/G	1.83E-07	22.78	0.1016	*GL7*/*GW7*/*SLG7*
	BIM	Hainan	2018	7	rs7_24279896	24279896	C/G	5.46E-07	17.96	−0.0061	*GL7*/*GW7*/*SLG7*
	BIM	Henan	2018	7	rs7_24505268	24505268	A/T	1.03E-05	10.42	−0.0740	–
*qGW8*	BIM	IRRI	2017	8	rs8_26573952	26573952	A/C	3.47E-09	22.78	0.0933	*GW8/OsSPL16*
	BIM	Hainan	2018	8	rs8_26573952	26573952	A/C	6.04E-08	17.96	0.0831	*GW8/OsSPL16*
	BIM	Henan	2018	8	rs8_26351058	26351058	C/G	5.61E-05	10.42	−0.0549	–
*qGLWR3*	DC1	Hainan	2017	3	AX-154083640	16806335	A/C	1.55E-06	63.35	0.1340	*GS3*
	DC1	Hainan	2018	3	AX-154083640	16806335	A/C	1.11E-06	59.60	0.0593	*GS3*
	BIM	IRRI	2017	3	rs3_16842769	16842769	C/T	5.96E-10	29.52	0.1453	*GS3*
	BIM	Hainan	2018	3	rs3_16842769	16842769	C/T	3.04E-08	17.95	0.0272	*GS3*
	BIM	Henan	2018	3	rs3_16842769	16842769	C/T	1.27E-05	11.98	0.1624	*GS3*
*qGLWR5*	DC1	Hainan	2017	5	AX-165092179	5364311	G/T	2.31E-09	63.35	0.1667	*GW5*/*qSW5*
	DC1	Hainan	2018	5	AX-165085603	5368362	C/T	8.29E-08	59.60	0.0046	*GW5/qSW5*
	BIM	IRRI	2017	5	rs5_4685451	4685451	C/G	1.04E-07	29.52	0.2049	–
	BIM	Hainan	2018	5	rs5_4685451	4685451	C/G	2.40E-07	17.95	0.0125	–
*qGLWR7*	BIM	IRRI	2017	7	rs7_24686632	24686632	A/G	7.88E-10	29.52	0.2320	*GL7*/*GW7*/*SLG7*
	BIM	Hainan	2018	7	rs7_24279896	24279896	C/G	6.46E-10	17.95	−0.0297	*GL7*/*GW7*/*SLG7*
	BIM	Henan	2018	7	rs7_24505268	24505268	A/T	4.29E-07	19.23	0.1819	*GL7*/*GW7*/*SLG7*
*qGLWR8*	BIM	IRRI	2017	8	rs8_26573952	26573952	A/C	1.22E-07	29.52	−0.1599	*GW8*
	BIM	Hainan	2018	8	rs8_26573952	26573952	A/C	3.09E-07	17.95	0.0237	*GW8*
*qGT1*	BIM	IRRI	2017	1	rs1_6640911	6640911	C/T	3.54E-06	22.47	−0.0311	–
*qGT3*	BIM	Henan	2018	3	rs3_17044632	17044632	A/G	8.04E-05	6.43	−0.0231	–
*qGT5*	DC1	Hainan	2017	5	AX-155076620	5420315	A/C	1.01E-05	30.87	−0.0367	*GW5*/*qSW5*
	DC1	Hainan	2018	5	AX-165092179	5364311	G/T	2.35E-06	33.68	−0.0009	*GW5*/*qSW5*
	BIM	Hainan	2018	5	rs5_5391586	5391586	A/C	8.39E-06	21.79	−0.0004	–
*qTGW3.1*	DC1	Hainan	2017	3	AX-154017324	3310128	A/G	9.51E-06	40.47	–	–
*qTGW3.2*	BIM	IRRI	2017	3	rs3_16996623	16996623	G/T	6.79E-09	31.31	1.0800	*GS3*
	BIM	Hainan	2018	3	rs3_16736681	16736681	G/T	4.71E-06	21.19	0.1362	*GS3*
	BIM	Henan	2018	3	rs3_16729992	16729992	G/C	4.78E-08	19.94	1.4490	*GS3*
*qTGW5*	DC1	Hainan	2017	5	AX-165092179	5364311	A/G	2.14E-06	40.49	−1.1514	*GW5*/*qSW5*
	DC1	Hainan	2018	5	AX-165092179	5364311	A/G	6.23E-06	40.80	−0.0938	*GW5*/*qSW5*
*qTGW8.1*	DC1	Hainan	2018	8	AX-115754523	861108	C/T	9.21E-05	40.47	0.1782	–
*qTGW8.2*	BIM	Henan	2018	8	rs8_26349349	26349349	G/T	1.94E-05	11.38	0.7771	–

*^a^Environment where the trial was conducted, ^b^Chromosome number, ^c^Physical position (bp), ^d^Major/minor allele, ^e^Percent phenotypic variance explained by the marker, ^f^Allele effect with respect to minor allele.*

Three QTLs, two on chromosome 3 and one on chromosome 7, were identified for GL (Figure 3). *qGL3.1* and *qGL3.2* were consistently detected in both of the DC1 and BIM populations across the three testing environments. These two closely localized QTLs were in different LD blocks (Supplementary Figure 4) and are therefore independent of each other. Phenotypic variance of *qGL3.1* ranged from 21.56 to 55.28%, whereas it ranged from 17.74 to 55.28% for *qGL3.2* (Table 1). Four significant SNPs in *qGL3.1*, one in the coding region and three located at 7.2 to 7.3 kb downstream of the *GS3* gene, were consistently identified in the BIM population in the three testing environments. A total of 10 significant SNPs of which 8 were in the intron, 1 in the coding region, and 1 at about 6.2 kb downstream of the *GS3* gene were identified significant in the DC1 population. Interestingly, significant non-synonymous SNPs AX-115826214 in the DC1 and rs3_16729992 in the BIM were within the coding region of *GS3* gene. *qGL7* was detected only in the BIM population, which explained 35.07 and 21.60% of the total phenotypic variance in IRRI 2017 and Hainan 2018, respectively. The peak SNP, rs7_24669663, in this locus was located at 5.2 kb upstream of the *GL7*/*GW7*/*SLG7* gene (Supplementary Table 3).

A total of three QTLs were detected on chromosomes 5, 7, and 8 for GW (Figure 3). *qGW5* was detected in the DC1 and explained 59.78 and 24.88% of the total phenotypic variance in Hainan 2017 and Hainan 2018, respectively. This QTL was also detected in the BIM population in all the three testing environments with the phenotypic variance explained ranging from 10.42 to 22.78%. In this locus, the two significant SNPs, AX-155172546 and AX-165092179, detected in the DC1 were within 1.2 kb upstream of the *GW5*/*qSW5* gene. *qGW7* and *qGW8* were detected only in the BIM population, both of which explained 22.78, 17.96, and 10.42% of the total phenotypic variation in IRRI 2017, Hainan 2017, and Hainan 2018, respectively. The peak SNP, rs7_24279896, in *qGL7* was located at 5.2 kb upstream of the *GL7*/*GW7*/*SLG7* gene. Moreover, the peak SNP, rs8_26505685, in *qGW8* loci was within the coding region of *GW8* (Table 1 and Supplementary Table 3).

Four QTLs located on chromosomes 3, 5, 7, and 8 were identified for GLWR (Figure 3). *qGLWR3* and *qGLWR5* explained very high proportions of the phenotypic variances in the DC1 population in Hainan 2017 (63.35%) and Hainan 2018 (59.60%). On the contrary, relatively low proportions of the phenotypic variations were observed in the BIM population ranging from 11.98 to 29.52% for *qGLWR3* and 17.95 to 29.52% for *qGLWR5*. It is noteworthy that *qGLWR3* was stably detected in both populations across all the three testing environments. In the DC1 population, eight, one, and one significant SNP/s were located in the intron, the coding region, and 6.2 kb upstream of the *GS3*, respectively. For the BIM population, four significant SNPs were within the *GS3* gene of which one SNP, rs_16729992, was located in the coding region and three SNPS were within 7.2 to 7.3 kb downstream of the gene. Moreover, two significant SNPs, AX-155172546 and AX-165092179, in *qGLWR5* were within 1.2 kb upstream of the *GW5*/*qSW5* gene. The QTLs *qGLWR7* and *qGLWR8* were both detected in the BIM population. *qGLWR7* explained 17.95–29.52% of the total phenotypic variances. The significant SNP, rs7_24669663, in this locus was within 5.2 kb upstream of the *GL7*/*GW7*/*SLG7* gene. *qGLWR8* explained 17.95–29.52% of the total phenotypic variance and it is noteworthy that the significant SNP, rs8_26505685, in this locus was within the coding region of the *GW8/OsSPL16* gene (Table 1 and Supplementary Table 3).

Three QTLs located on chromosomes 1, 3, and 5 were detected for GT. *qGT1* and *qGT3* were detected only in the BIM population in IRRI 2017 and in Henan 2018, respectively. *qGT1* explained moderate phenotypic variance (22.47%), whereas *qGT3* had relatively low phenotypic variance (6.43%). *qGT5* was identified in both populations and explained 21.79–33.68% of the total phenotypic variation. The peak SNP in this QTL, AX-165092179, was located at 1.2 kb upstream of the *GW5*/*qSW5* gene (Table 1 and Supplementary Table 3).

A total of five QTLs, two on chromosomes 3 and 8 and one on chromosome 5, were detected for TGW. *qTGW3.1* was detected only in the DC1 population in Hainan 2017 and accounted for 40.47% of the total phenotypic variance. *qTGW3.2* was detected only in the BIM population and explained 19.94–31.31% of the total phenotypic variance. Four significant SNPs in the *qTGW3.2* locus were in the untranslated and coding region of *GS3*. *qTGW5* was detected only in DC1 population with 40.49 and 40.80% of the phenotypic variance in Hainan 2017 and Hainan 2018, respectively. Two significant SNPs, AX-155172546 and AX-165092179, in this QTL were within 1.2 kb upstream of the *GW5*/*qSW5* gene. *qTGW8.1* was detected only in the DC1 population in Hainan 2018 and accounted for 40.47% of the total phenotypic variance, whereas *qTGW8.2* was detected in the BIM population in Henan 2018 and accounted for 11.38% of the total phenotypic variance (Table 1 and Supplementary Table 3).

### Potential Candidate Genes for Promising QTLs

Based on both genetic background independency and stability across different environments, four QTLs on chromosomes 3 and 5 were considered promising (Figure 3). Potential candidate genes in these QTLs regions were narrowed down to a total of four (Table 2) through literature searches and by considering only those with significant non-synonymous SNPs in the coding region. Two potential candidate genes, LOC_Os03g29630 (*ulp1*) and LOC_Os03g29810 (*OsClp6*), were identified for *qGL3.1* and *qGLWR3*. Interestingly, *OsClp6* was identified in both of the DC1 and BIM populations across all the three testing environments. Two potential genes, LOC_Os05g08850 (putative cytochrome P450) and LOC_Os05g10620 (NAC domain containing protein 75), were identified for *qGW5* and *qGLWR5*.

**TABLE 2:** Potential candidate genes from promising QTLs detected for the grain size-related traits.

QTLs	Gene ID	Gene annotation	SNP marker	Position	SNP
*qGL3.1*, *qGLWR3*	LOC_Os03g29630	ulp1 protease family protein, putative, expressed	AX-115855710	16890773	A/G
	LOC_Os03g29810	OsClp6—Putative Clp protease	AX-115799679	16996623	G/T
			AX-115815041	16997653	C/T
			AX-165099608	16993369	C/T
			rs3_16996623	16996623	G/T
*qGW5*, *qGLWR5*	LOC_Os05g08850	Cytochrome P450, putative, expressed	rs5_4885582	4885582	C/A
			rs5_4886725	4886725	T/G
	LOC_Os05g10620	No apical meristem protein, putative,	AX-115819199	5785251	C/A
		expressed/NAC domain containing protein 75	AX-154635971	5799150	C/A

## Discussion

### Phenotypic Variation and Trait Correlation

Wide phenotypic variation was observed for all the traits in all the testing environments relative to the founder lines, suggesting the formation of transgressive segregants. Transgressive segregation is of interest for breeders as it provides breeding materials best use for crop improvement. The wide variability for grain size-related traits among the MAGIC lines under study will allow breeders to select superior lines with improved grain size. The high variation observed also suggests that both of the DC1 and BIM populations can be effectively utilized in finding allelic variants responsible for grain size differences.

In the present study, positive and strong correlations were observed between GL and TGW, GT, and GW, which was consistent with the previous studies (Tan et al., 2000; Wang et al., 2012b). This suggests that GL has the largest effect on grain weight compared with other grain size-related traits (Rui and Zhao, 1983; Lin and Wu, 2003; Xing and Zhang, 2010), whereas GW contributes more on GT. Very weak and positive correlation between TGW and GLWR was observed in the two MAGIC populations across the three testing environments. This result was consistent with the findings of Qiu et al. (2015), but different with the result of Xu et al. (2015b).

### Population Structure and Whole Genome Pattern of LD Decay

One of the advantages of the MAGIC populations is that they are homogeneous without population structure. Indeed, no substructure was observed for the MAGIC populations in rice (Bandillo et al., 2013; Meng et al., 2016), tomato (Pascual et al., 2015), wheat (Huang et al., 2012a), and barley (Sannemann et al., 2015). In this study, no subpopulation was observed in the BIM population, whereas obvious substructure was observed in the DC1. By carefully inspecting the genotype of the DC1 lines, it was found that substructuring was caused by the presence of a few exceptionally similar lines. Removing these lines resulted to no substructure. It is therefore important in the GWAS that the analysis of population structure must be done prior to association regardless of the type of the populations.

The LD decay of the MAGIC populations under study ranged from 1.70 to 2.25 Mb. This distance was longer compared to *indica* rice germplasm panels previously reported by Huang et al. (2012b); Mcnally et al. (2009), and Zhao et al. (2011), which ranged from 100 kb to 1 Mb. This is not surprising due to many historical recombination events of *indica* rice germplasm panels, which contributed to its rapid LD decay. Thus, varying patterns of LD likely reflect the breeding histories and the origins of the germplasm panel used (Flint-Garcia et al., 2003).

### Genetic Background and Environment Effects on QTLs Detected

Two of the most important considerations in QTL detection studies are the genetic background effect and QTL-by-environment interaction (QEI), i.e., whether the reported QTLs are robust across different populations and environments. In the present study, the number of QTLs detected significantly varied between two MAGIC populations and across environments, suggesting that GWAS results were indeed highly influenced by both the population’s genetic background and testing environment. Out of the 18 QTLs identified, only 6 QTLs (*qGL3.1, qGL3.2, qGW5, qGLWR3, qGLWR5*, and *qGT5*) were consistently detected from the two populations under study. Interestingly, *qGL3.1, qGL3.2, qGW5*, and *qGLWR3* were stably expressed across all the three environments. These QTLs are therefore important in marker-assisted breeding to improve grain size. *qGW7, qGW8, qGLWR7*, and *qTGW3.2* were also stably expressed in all the three testing environments but were detected only in the BIM population. The QTLs for GT (*qGT1, qGT3*, and *qGT5*) and TGW (*qTGW3.1, qTGW5, qTGW8.1*, and *qTGW8.2*) were detected in only one of the testing environments, suggesting that these two traits were more sensitive to the environment leading to phenotypic plasticity. Phenotypic plasticity is a result of the interaction between QTLs and environment at the molecular level, and therefore, the genotype displays good trait performance only in a specific environment (Sultan, 2000). In this study, inconsistency of the QTLs detected could also be attributed to the threshold level used to declare significant MTAs. For instance, *qGL7* and *qGLWR8* were not identified in one of the testing environments (Henan 2017) because the *p*-values of SNPs were slightly higher (−log *p* = 1.31E-04 to 1.51E-04) than the applied critical threshold (−log *p* < 1.0E-04). In many QTL detection studies either through linkage or association mapping, inconsistency of the QTLs detected could be due to the type II error arising from the significant threshold level or to the true differential trait expression across environments.

### BIM Has Higher Mapping Power and Resolution Than DC1

Nine QTLs (*qGT1*, *qGT3*, *qTGW3.2*, *qGL7*, *qGW7*, *qGLWR7*, *qGW8*, *qGLWR8*, and *qTGW8.2*) were identified only in the BIM population. This is at least partially due to the higher allelic and phenotypic diversity of the BIM population offered by the larger number of parental accessions used compared with the DC1 population. Hence, multiple parents will ensure that several QTLs for the trait of interest segregating within the population could be identified (Kover et al., 2009; Huang et al., 2015). Moreover, high recombination events in the BIM population implies higher genetic mapping resolution, either coarse mapping with low marker densities on early generation lines or fine mapping with high marker densities on late generation lines (Mackay and Powell, 2007). The effect of population size on the GWAS is also well known (Wang et al., 2012a). The larger size of the BIM population (Supplementary Table 2) also contributed to its higher mapping power and resolution. For a QTL to be effectively detected in a small population, it must be in high LD with the tested markers. Furthermore, increasing population size is necessary in detecting loci with low MAF since the power to detect association is a function of allele frequency (Myles et al., 2009). In our study, the size of DC1 population was small and the *GL7* and *GW8* genes were missed out, since the SNP markers in strong LD with these genes had low MAF and were removed prior to association analysis. Nevertheless, loci with large effects (*qGL3.1*, *qGW5*, *qGLWR3*, and *qGLWR5*) detected in the BIM population were also detected in the DC1 population. Therefore, if the MAF is high enough, major loci controlling the trait of interest would still be detected regardless of low population size.

### Potential Candidate Genes Controlling Grain Size Were Identified

Two potential candidate genes, the ubiquitin-like protein 1 (*ulp1*) and *OsCLP6*, were identified for QTLs *qGL3.1* and *qGLWR3.* The *upl1* (LOC_Os03g29630) is likely to be involved in the ubiquitin-proteasome proteolytic pathway controlling grain size. Several ubiquitin-proteasome pathway-related genes were previously characterized and involved in ubiquitin-mediated control of seed size. The ubiquitin-proteasome pathway has been recently shown to play a crucial role in regulating seed size in different crops (Li and Li, 2016). In rice, the protein of unknown function encoded by *GW5* has been suggested to be involved in the pathway through its interaction with polyubiquitin (Weng et al., 2008). The RING-type E3 ubiquitin ligase encoded by *GW2* positively regulates GW and grain weight by restricting cell division (Song et al., 2007). *HGW*, which encodes a novel plant specific ubiquitin-associated (UBA) domain protein, functions as a key upstream regulator of GW (Li et al., 2012). The rice deubiquitinase gene *OsOTUB1*/*WTG1* encodes an otubain-like protease and affects grain size (Huang et al., 2017; Liu et al., 2018). Recently, *LG1* encoding for ubiquitin specific protease 15 (*OsUBP15*) was reported to function as a positive regulator of GW in rice (Shi et al., 2019). The other potential candidate gene, *OsCLP6* (LOC_Os03g29810), encoding a putative chitinase-like protein (*Clp*) protease homolog was identified in both populations across all the three testing environments. Chitinases are classic pathogenesis-related proteins involved in plant growth and development regulation with the first genetic evidence observed in the Arabidopsis *AtCTL1* gene (Graham and Sticklen, 1994; Zhong et al., 2002). In rice, secreted chitinase-like proteins (*OsCLP*) have been shown to play a pivotal role in root and shoot growth by regulating the intracellular calcium concentrations. Interestingly, Wu et al. (2017) reported that *OsCLP* negatively regulates rice GL with the seeds of CLP overexpression lines being shorter and rounder than those of the wild type and the *osclp* mutant.

Two genes, putative cytochrome P450 (*Cyp*/LOC_Os05g08850) and putative no apical meristem (NAC) gene (LOC_Os05g10620), were identified as potential candidate genes for the QTLs *qGW5* and *qGLWR5*. Cytochrome proteins (CYPs) have been reported to play a crucial role in a variety of biosynthetic pathways including brassinosteroid (BR) signaling. In rice, *CYP724B1* encoded by the *D11* gene showed homology to enzymes involved in BR biosynthesis. Loss of function of *D11* impaired BR biosynthesis and ultimately resulted to reduction in GL (Tanabe et al., 2005). BR-mediated grain size control in rice has been reported for several QTLs/genes including *GS5* (Li et al., 2011), *qGL3*/*GL3.1* (Qi et al., 2012; Zhang et al., 2012), *GW5*/*qSW5* (Wan et al., 2008; Weng et al., 2008; Liu et al., 2017), *GS2* (Che et al., 2015), and *GS9* (Zhao et al., 2018). Defects in BR biosynthesis produce smaller seeds, but it is still uncertain how BRs promote seed growth (Hong et al., 2002, 2003). It is noteworthy that several CYP family members are involved in seed size control, including the Arabidopsis *CYP78A5, CYP78A6*, and *CYP78A9* (Adamski et al., 2009; Fang et al., 2012), and soybean *CYP78A72* and *CYP78A10* (Wang et al., 2015b; Zhao et al., 2016), which all act as positive regulators of grain size, whereas the rice *CYP78A13* and *CYP704A3* (Xu et al., 2015a; Tang et al., 2016) act as negative regulators. NAC genes have been implicated in the control of seed size via the control of various stages of seed development (Agarwal et al., 2011). Three NAC genes, *ONAC020* (LOC_Os01g01470), *ONAC026* (LOC_Os01g29840), and *ONAC023* (LOC_Os02g12310), were reported to be highly upregulated during seed development and were strongly associated with rice grain size from the analysis of sequence variations in the upstream regulatory region (Mathew et al., 2016).

## Conclusion

Many grain size-related genes have been identified and cloned in the last decades; however, their molecular roles and their interaction between different signaling pathways are still fragmented. Therefore, identification and functional characterization of new genes are of significance. Using two MAGIC populations tested in three environments, the present study provided additional insight on the genetic architecture of rice grain size. Identification of QTLs with genetic background independency and environment stability is essential because grain size-related traits show phenotypic plasticity. The QTLs and the significant SNPs identified in this study, particularly those that exhibited genetic background independency and environment stability, will be useful for breeding *indica* rice to improve grain size. Moreover, the potential candidate genes reported are also important targets for future functional characterization studies to fill up the gaps and/or build up genetic framework of signaling pathways regulating grain size in rice. Methodologies such as gene editing and transferred DNA insertion mutant screens could be used to validate the effect of these genes and their functional variants. It is also noteworthy that populations derived from multiparent crosses provide more phenotypic and allelic diversity than the conventional biparental populations and have lesser confounding effects in terms of population structure and genetic relatedness than natural populations. The present study proved that the BIM population was generally better than the DC1 population.

## Data Availability Statement

All datasets generated and analyzed for this study are included in the article/Supplementary Material.

## Author Contributions

YL, GY, and LG designed the experiment. KP, YZ, and YL performed all the phenotypic evaluations. KP performed the data analysis and drafted the manuscript. YL and GY edited the manuscript. All authors approved the content of this manuscript.

## Conflict of Interest

The authors declare that the research was conducted in the absence of any commercial or financial relationships that could be construed as a potential conflict of interest.
