# Supplementary material for: Genome-Wide Association Study of Grain Size Traits in Indica Rice Multiparent Advanced Generation Intercross (MAGIC) Population
Source: Front Plant Sci. 2020 Apr 24;11:395. doi: 10.3389/fpls.2020.00395 (PMC7193545; doi:10.3389/fpls.2020.00395)
Supplement: Supplementary file 2 [file Data_Sheet_2.pdf]

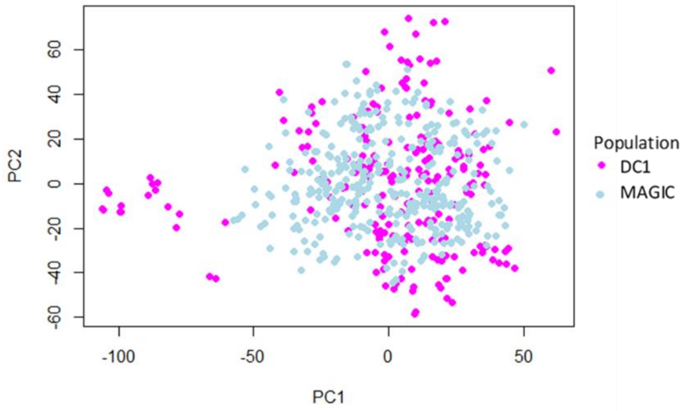

Supplementary Figure 1 Population structure of DC1 and 8-way populations based on PCA.

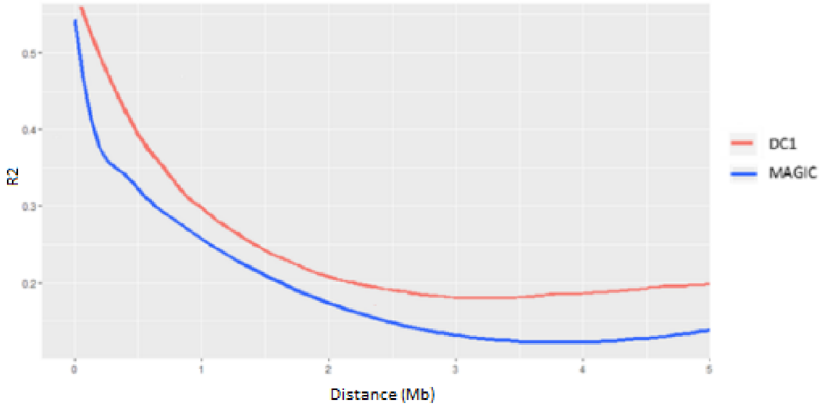

Supplementary Figure 2 Whole genome linkage disequilibrium decay of DC1 and 8-way populations.

(A)
GS3

(A)
GS3

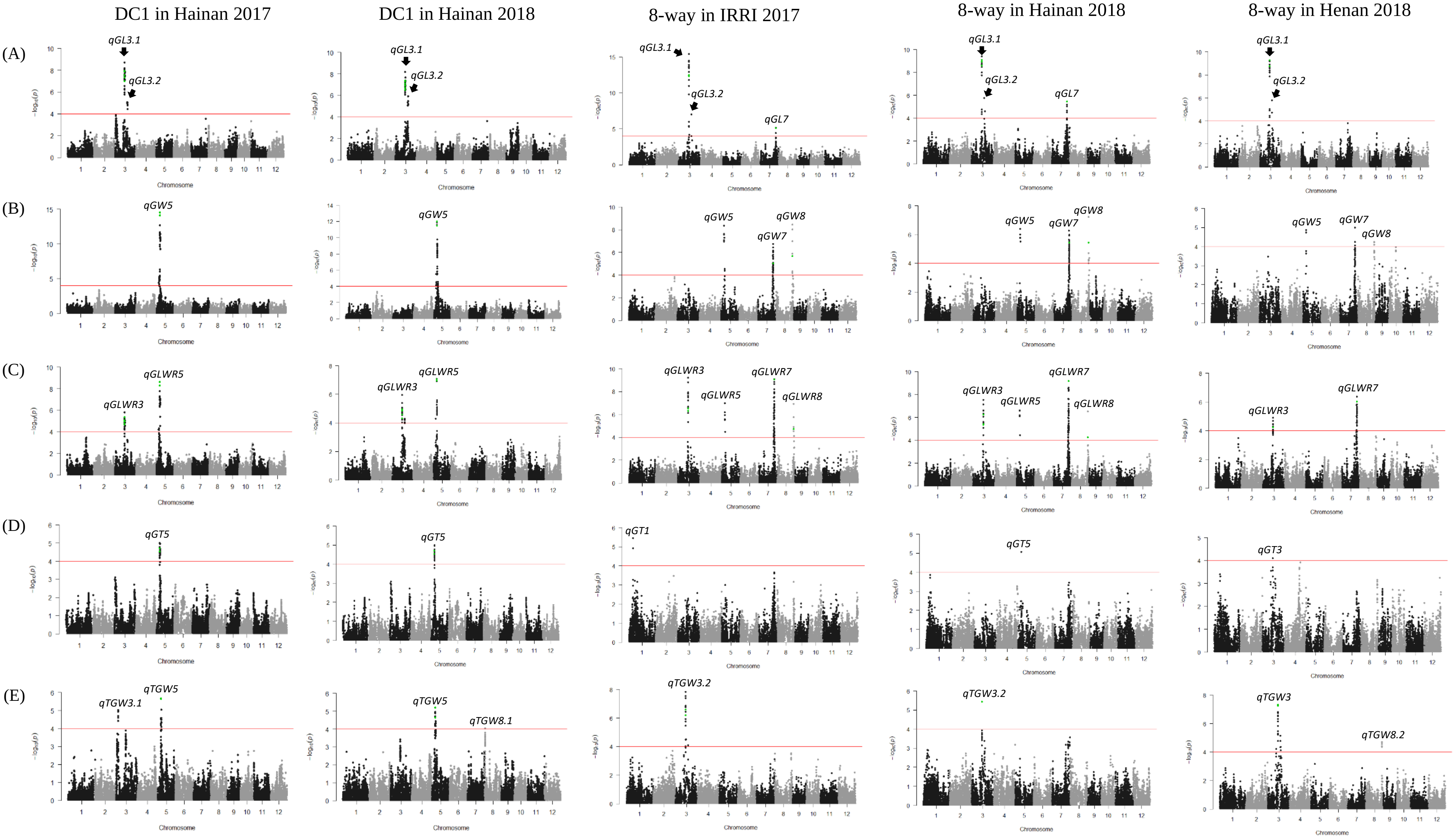
DC1 in Hainan 2017
DC1 in Hainan 2018
8-way in IRRI 2017
8-way in Hainan 2018
8-way in Henan 2018
(A)
(B)
(C)
(D)
(E)
qGL3.1
qGL3.2
qGL7
qGW5
qGW7
qGW8
qGLWR3
qGLWR5
qGLWR7
qGLWR8
qGT1
qGT3
qGT5
qTGW3
qTGW3.1
qTGW3.2
qTGW5
qTGW8.1
qTGW8.2
Chromosome

Supplementary Figure 3 Genome-wide Manhattan plots for (A) Grain length, (B) grain width, (C) grain length-width ratio, (D) grain thickness, and (E) thousand grain weight in DC1 and 8-way measured in different environments and across years. Dots in green color corresponds to SNPs of previously cloned genes (*GS3*, *GW5/qSW5*, *GL7*/*GW7*/*SLG7*, and *GW8/OsSPL16* on chromosomes 3, 5, 7 and 8, respectively).

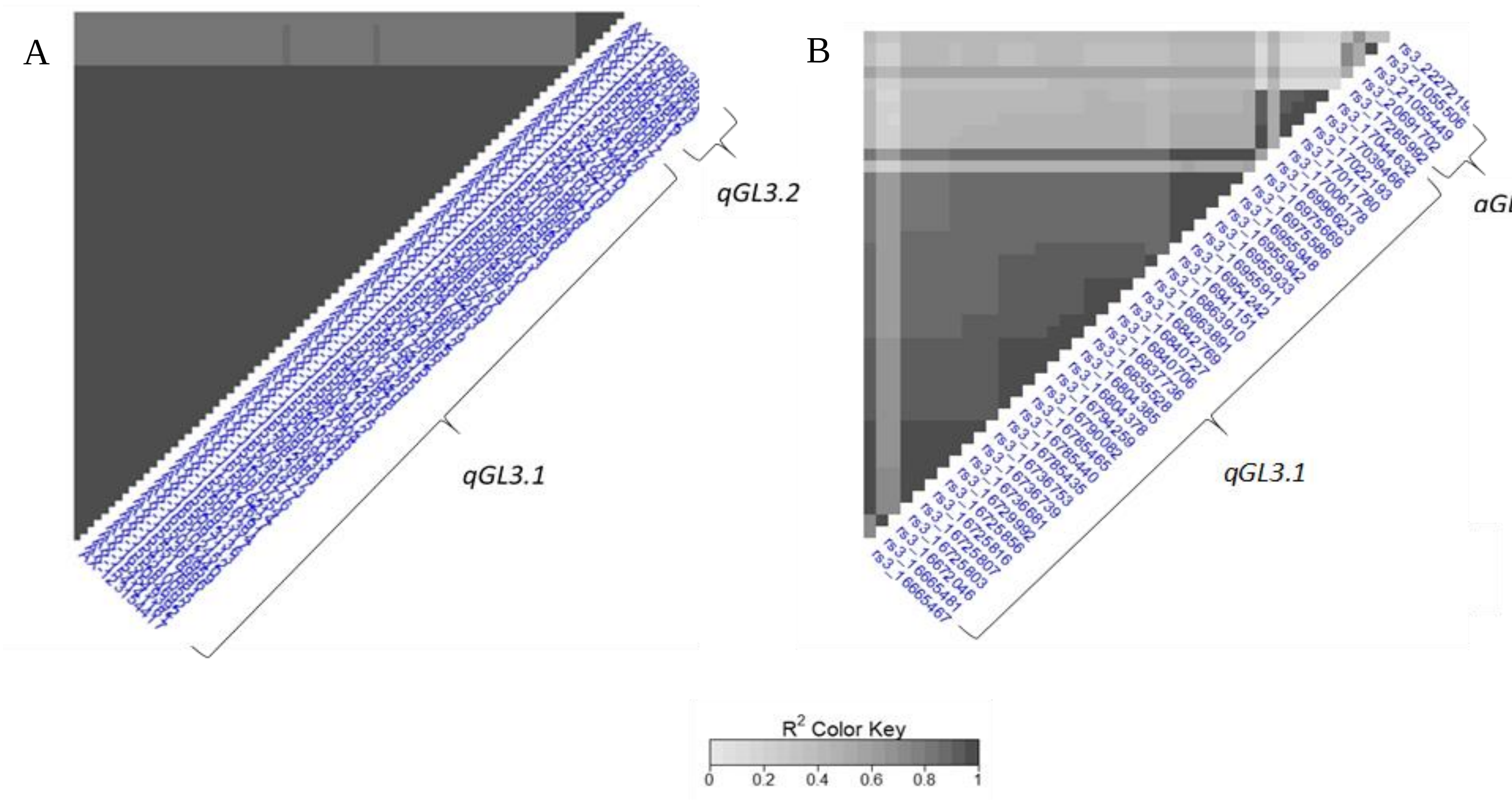

Supplementary Figure 4 LD blocks for *qGL3.1* and *qGL3.2* detected in (A) DC1 and (B) 8-way populations.
